# Graph-Based Topological Embedding and Deep Reinforcement Learning for Autonomous Voltage Control in Power System

**DOI:** 10.3390/s25030733

**Published:** 2025-01-25

**Authors:** Hongtao Wei, Siyu Chang, Jiaming Zhang

**Affiliations:** College of Information Engineering, Wuhan University of Technology, Wuhan 430070, China; weiht@whut.edu.cn

**Keywords:** deep reinforcement learning (DRL), Graph Convolutional Network (GCN), soft actor-critic (SAC), voltage control, load shedding

## Abstract

With increasing power system complexity and distributed energy penetration, traditional voltage control methods struggle with dynamic changes and complex conditions. While existing deep reinforcement learning (DRL) methods have advanced grid control, challenges persist in leveraging topological features and ensuring computational efficiency. To address these issues, this paper proposes a DRL method combining Graph Convolutional Networks (GCNs) and soft actor-critic (SAC) for voltage control through load shedding. The method uses GCNs to extract higher-order topological features of the power grid, enhancing the state representation capability, while the SAC optimizes the load shedding strategy in continuous action space, dynamically adjusting the control scheme to balance load shedding costs and voltage stability. Results from the simulation of the IEEE 39-bus system indicate that the proposed method significantly reduces the amount of load shedding, improves voltage recovery levels, and demonstrates strong control performance and robustness when dealing with complex disturbances and topological changes. This study provides an innovative solution to voltage control problems in smart grids.

## 1. Introduction

Modern power systems are typical examples of nonlinear complex systems, and their stable operation heavily depends on maintaining bus voltages within standard ranges. However, as the scale of power systems continues to expand, along with the large-scale integration of renewable energy and the widespread adoption of electric vehicles, grid security and stability face unprecedented challenges due to increasing uncertainties. In practice, generator outages or tie-line faults may lead to local active power imbalances, causing a drop in grid frequency and triggering the low-frequency protection mechanisms of generators. This cascading effect can further result in the disconnection of a large number of generators, potentially leading to system collapse. In recent years, multiple large-scale blackouts worldwide have underscored the existence and devastating impact of this vicious cycle. Therefore, adopting rapid and effective measures to reduce active power imbalances during the early stages of power system failures is critical for preventing grid frequency collapse and ensuring stable system operations. When system load demand exceeds generation capacity, proactive or reactive load shedding has been proven as an essential strategy for maintaining power system stability [1].

Traditional voltage control methods primarily rely on historical experience and offline studies [2,3,4,5,6], which exhibit significant limitations in addressing the dynamics and stochastic variations of complex systems. These methods often fail to fully exploit the system’s adjustment potential due to their conservative nature, or struggle to adapt to rapidly changing system states due to high associated risks. For example, Li et al. [2] proposed an emergency control method based on optimal control, which improved computational efficiency but exhibited limited adaptability due to its dependence on precise models. Yang et al. [3] developed a real-time adaptive load-shedding strategy optimized with fuzzy logic controllers, which performed well in simulations but faced challenges in practical implementation due to its complexity. Jianjun et al. [4] optimized load-shedding locations and magnitudes using wide-area measurement information but required further validation of its dynamic adaptability under multiple disturbance scenarios. Usman et al. [5] utilized the evolutionary particle swarm optimization (EPSO) algorithm to enhance load-shedding accuracy and economic efficiency but encountered high computational complexity. Lozano et al. [6] introduced a decision tree algorithm based on PMU data, which improved flexibility in load shedding but exhibited limited performance in complex fault scenarios. In summary, traditional methods show notable deficiencies in addressing the randomness and dynamics of modern power systems, necessitating the development of more efficient and intelligent control strategies.

The swift progress of artificial intelligence (AI) in recent years has opened up new opportunities for addressing complex power grid control challenges. Reinforcement learning (RL), a key branch of AI, has gradually been applied in the power industry due to its advantages in dynamic decision-making and optimization [7]. By iteratively optimizing policies in complex environments, RL provides intelligent real-time control capabilities for power systems. Particularly in critical problems, such as dynamic load shedding and voltage control, deep reinforcement learning (DRL) has demonstrated significant potential for addressing the complexity of modern power systems, owing to its powerful state representation and action optimization capabilities. DRL has thus become a key research direction in grid control.

Existing studies have explored the application of DRL in voltage control. For instance, Chen et al. [8] proposed a distributed deep reinforcement learning (DDRL) approach, which achieved coordinated bus agent operation through centralized training and decentralized execution, demonstrating excellent performance in real-time load shedding and voltage stability control. Zhang and Yue [9] developed a cooperative multi-agent DRL (MADRL) method with an attention mechanism, improving agent collaboration efficiency and achieving more precise distributed load shedding. However, these methods still face challenges in scalability and computational efficiency. The policy-adaptive reinforcement search (PARS) algorithm proposed in [10] improved training efficiency and robustness, addressing large-scale load-shedding tasks in the IEEE 300-bus system but lacked sufficient utilization of grid topology characteristics. Zhang et al. [11] introduced the PLASE method based on a parallel system framework, which enhanced load-shedding and voltage control through agent performance evaluation and self-evolution but did not fully explore the potential value of topology information for optimizing control strategies. Yan and Xu [12] proposed a DRL-based model-free approach for load frequency control, which optimized the dynamic response speed but exhibited limited research on its adaptability for voltage control.

In conclusion, although existing studies have made progress in voltage control for load shedding, notable challenges remain. On one hand, most methods fail to fully exploit grid topology characteristics, overlooking dynamic interactions between nodes and the potential value of higher-order topology information, which limits the decision-making capabilities of agents in complex networks. On the other hand, current strategies lack sufficient adaptability to the stochastic and rapidly changing operating conditions of power systems, making it difficult to achieve efficient and robust control under diverse scenarios. To address these challenges, this paper proposes a joint optimization method combining Graph Convolutional Networks (GCNs) and DRL. By leveraging grid topology characteristics and optimizing dynamic control strategies, this method enhances the precision and efficiency of load-shedding control. Additionally, the soft actor-critic (SAC) algorithm is employed to improve policy robustness and stability. This approach not only addresses the limitations of existing studies but offers an innovative solution for voltage stability control in complex dynamic environments.

The main contributions of this study are as follows:

- Proposing an intelligent voltage control method combining GCNs and DRL, capable of efficiently regulating voltages by fully utilizing grid topology information.
- Employing GCNs for graph embedding to extract topological relationships among nodes, providing DRL with more precise and discriminative state representations.
- Designing an optimized load-shedding strategy and validating its effectiveness and robustness under various topological change scenarios.

This remainder of this paper is organized as follows: Section 2 outlines the concepts of reinforcement learning and related research on power grid emergency control modeling, laying a theoretical foundation for the subsequent methods. Section 3 describes the proposed methods in detail, including the GCN for feature extraction, the DRL algorithm for action decision optimization, the overall method combining GCNs and DRL, and the Markov decision process (MDP) modeling of power grid emergency control. Section 4 presents the simulation results, including the simulation settings, the training and testing performance of the model, and the analysis of the optimized load shedding strategy. Section 5 summarizes the research results of this paper and looks forward to future research directions.

## 2. Overview of Reinforcement Learning and Power Grid Emergency Control Modeling

This chapter aims to lay the theoretical foundation and modeling basis for the proposed voltage control method based on DRL. First, the core theory of RL—MDP—is systematically reviewed, and its advantages in modeling dynamic optimization problems are analyzed to provide theoretical support for the design of intelligent control strategies. Second, a mathematical model tailored for power grid emergency control is constructed, considering the operational characteristics of power grids. The core variables, constraints, and their relationships with the system’s dynamic behavior in the context of voltage control are clearly defined, offering a scientific basis for practical model applications.

### 2.1. Markov Decision Process in Reinforcement Learning

In RL, the environment is modeled as a partially observable Markov decision process (MDP). The MDP is a mathematical framework for describing decision-making problems with stochastic dynamics, defined by the following components:

- State space (S): The state space encompasses all possible states that the environment can be in. Each state s∈S provides a comprehensive description of the environment’s current condition and may include features across multiple dimensions.
- Action space (A): The action space refers to the complete set of all possible actions that an agent can take. Each action a∈A represents the agent’s control input to the environment in the current state.
- Transition function (P:S×A→S): The transition function specifies the probability of transitioning to a new state $s_{t+1}$ given the current state $s_{t}$ and action $a_{t}$.
- Reward function ($R_{t}$): The reward function defines the immediate reward received after taking action $a_{t}$ in state $s_{t}$.
- Discount factor (γ∈[0,1]): The discount factor indicates the importance of future rewards compared to immediate rewards, balancing short-term and long-term goals.

At each time step t, the agent observes the environment’s current state $s_{t}\in S$ and receives a reward signal $r_{t}\in R$ accordingly. Based on its current policy $\pi (a_{t}|s_{t})$, the agent selects an action $a_{t}$, influencing the environment and triggering a state transition.

The objective of RL is to optimize the policy $\pi (a_{t}|s_{t})$ to select the optimal action in a given state, thereby maximizing the agent’s long-term cumulative reward. The cumulative reward $G_{t}$ is the weighted discounted sum of future rewards, mathematically defined, as shown in Equation (1).

(1) $$G_{t}=\sum_{k=0}^{\infty }\gamma^{k}r_{t+k+1}$$

where γ is the discount factor; $r_{t+k+1}$ is the reward at time t+k+1; and $G_{t}$ quantifies the total value of future rewards from the current state t.

### 2.2. Power Grid Emergency Control Modeling

In large-scale power systems, the emergency control issue is a typical problem of highly nonlinear and non-convex optimal decision-making. Its core objective is to employ appropriate control measures to maximize the restoration or maintenance of system stability while satisfying system operational constraints. To formally describe this problem, it can be modeled as an optimization problem, as shown in Equations (2)–(7).

(2) $$\min\int_{T_{0}}^{T_{c}}C(x_{t},y_{t},a_{t})dt$$

(3) $$s.t.\;{\dot{x}}_{t}=f(x_{t},y_{t},e_{t},a_{t})$$

(4) $$0=g(x_{t},y_{t},e_{t},a_{t})$$

(5) $$x_{t}^{\min}\leq x_{t}\leq x_{t}^{\max},\forall t\in [T_{0},T_{c}]$$

(6) $$y_{t}^{\min}\leq y_{t}\leq y_{t}^{\max},\forall t\in [T_{0},T_{c}]$$

(7) $$a_{t}^{\min}\leq a_{t}\leq a_{t}^{\max},\forall t\in [T_{0},T_{c}]$$

Here, $x_{t}$ refers to the dynamic state variables of the power grid, primarily including physical quantities related to the grid’s dynamic characteristics, such as the angles and speeds of generator rotors. $y_{t}$ denotes the algebraic state variables of the grid, which typically include the voltage magnitudes and phase angles at the grid nodes. These variables describe the steady-state power flow distribution and reflect the instantaneous operating state of the power grid. $a_{t}$ encompasses the emergency control variables of the grid, such as load shedding. $e_{t}$ denotes possible unforeseen events or disturbances in the grid, such as short-circuit faults, line trips, or load fluctuations. These events are external disruptions that the system may encounter during operation. C(.). represents the cost function of emergency control in the power grid, which is the optimization objective for grid emergency control. $T_{0}$ and $T_{c}$ denote the time range.

Specifically, Equation (3) specifically outlines the dynamic behavior of different components within the power grid, reflecting their time-varying characteristics through differential equations. Equation (4) outlines the algebraic constraints among generators, transmission, and loads lines within the grid, capturing the network coupling relationships among these components, such as power balance and voltage constraints. Equations (5)–(7) define the operational limits and safety constraints for dynamic state variables, algebraic state variables, and control variables throughout the entire time range. These constraints collectively ensure the dynamic stability and safety of the grid during operation while providing a rigorous mathematical description for emergency control.

The emergency control problem mentioned above can be represented as an MDP and resolved using RL methods. The transition of environmental states from time t to t+1 is determined by the system of differential Equation (3) and algebraic Equation (4). The reward function $r_{t}$ depends on $x_{t}$, $y_{t}$ and $a_{t}$, with its specific form defined in Equation (8).

(8) $$r_{t}=h(x_{t},y_{t},a_{t})$$

The reward function $r_{t}$ should include the cost function C(.) as defined in Equation (2) and introduce penalty terms for violations of any constraints specified in Equations (5)–(7). The specific definition of the reward function, tailored to the requirements of the control strategy, will be elaborated in detail in Section 3.4.

## 3. Methodology

In this chapter, we systematically introduce the theoretical foundations of GCNs and the SAC algorithm. Based on these foundations, we propose a joint optimization method that combines GCNs and the SAC. Furthermore, we provide a detailed explanation of the MDP modeling process for implementing emergency control in power grids through load-shedding strategies. The core content of this chapter focuses on three main aspects: feature extraction, intelligent control strategy design, and problem modeling. These areas are analyzed comprehensively to elucidate the specific implementation details of the proposed method, providing robust theoretical and technical support for subsequent simulation validation and performance evaluation.

### 3.1. Graph Convolutional Networks for Feature Extraction

The topology of a power network can be abstracted as a complex graph, where nodes represent buses in the grid and edges represent the branches connecting these buses. The graph structure is defined by a set of nodes and edges, which are typically represented mathematically in the following two ways:

- Feature matrix (X): The feature matrix is an n×d matrix, where n is the number of nodes in the graph, and d is the dimensionality of features for each node. The i-th row of the matrix, $x_{i}$, represents the attribute information of node i such as voltage magnitude.
- Adjacency matrix (A): The adjacency matrix is an n×n matrix that encodes the graph’s connectivity structure. If there exists an edge between node i and node j, then $A_{ij}=1$; otherwise, $A_{ij}=0$.

In a GCN, the network takes the feature matrix X as input and propagates information through the adjacency matrix A to aggregate features, ultimately generating node-level output representations H. The output matrix H is an $n\times f^{\prime }$ matrix, where $f^{\prime }$ represents the dimensionality of the output features for each node. The core mechanism of a GCN lies in leveraging the adjacency matrix to perform weighted aggregation on the feature matrix. Through multiple layers of nonlinear transformation functions $f\left(.\right)$, it captures the local connectivity patterns in the graph and learns high-dimensional representations of the nodes [13]. The layer-wise propagation rule of a GCN is defined, as shown in Equation (9).

(9) $$H^{l+1}=f(H^{l},A)=\sigma \left\{{\hat{D}}^{-\frac{1}{2}}\hat{A}{\hat{D}}^{-\frac{1}{2}}H^{l}W^{l}\right\}$$

where l represents the number of layers in the network, $H^{l}$ is the node feature matrix at the l-th layer, with $H^{0}=X$, $\hat{D}$ is the degree matrix of $\hat{A}$, where $\hat{A}=A+I$,${\hat{D}}_{ii}=\sum_{j=1}^{n}{\hat{A}}_{ij}$, $W^{l}$ is the trainable weight matrix at the l-th layer, and σ(.) is the activation function. 

To capture the dynamic characteristics of voltage variations, this study selects bus voltage as the node feature for the GCN. Specifically, for each bus i in the power network, the most recently observed voltage values over a historical time window of length T are stacked into a vector to form the feature representation of node i at time t (Equations (10) and (11)).

(10) $$x_{i}^{\left(t\right)}=[v_{i}^{\left(t-T+1\right)},v_{i}^{\left(t-T+2\right)},\ldots ,v_{i}^{\left(t\right)}{]}^{\prime }$$

(11) $$X_{t}=[x_{1}^{\left(t\right)},x_{2}^{\left(t\right)},\ldots ,x_{n}^{\left(t\right)}{]}^{\prime }$$

### 3.2. DRL Algorithm for Action Decision Optimization

In addressing voltage instability issues induced by fault-induced delayed voltage recovery (FIDVR), selecting an appropriate DRL algorithm for policy optimization is crucial. This study adopts the SAC algorithm, an off-policy actor-critic model based on the maximum entropy reinforcement learning framework [14,15,16]. The SAC aims to learn a highly stochastic policy that can still effectively accomplish the target task. The core idea of the SAC is to incorporate policy entropy (a measure of policy randomness) into the reward signal. By optimizing an objective function that includes an entropy term, the SAC encourages the policy to achieve a dynamic balance between exploration and exploitation. This characteristic makes it well-suited for handling the dynamic changes in complex environments and prevents the policy from converging to suboptimal solutions due to excessive determinism.

Compared to other classical DRL algorithms, the SAC offers significant advantages:

- Compared to off-policy algorithms: Unlike the deep deterministic policy gradient (DDPG) algorithm [17,18] and the twin delayed deep deterministic policy gradient (TD3) algorithm [19,20], the SAC introduces the maximum entropy framework, significantly improving sampling efficiency and exploration capabilities.
- Compared to on-policy algorithms: In contrast to methods such as proximal policy optimization (PPO) [21], the SAC’s off-policy update mechanism reduces the sample correlation problem and achieves higher training efficiency.

Based on these features, this study applies the SAC for action decision optimization, aiming to address the complexity of dynamic power grid environments and the challenges of high-dimensional continuous action spaces.

The training objective of the SAC algorithm is to simultaneously maximize the expected return and entropy. Its objective function is defined by Equation (12).

(12) $$J(\pi )=E_{(s_{t},a_{t})∼\rho_{\pi }}[\sum_{t=0}^{T}\gamma^{t}(r(s_{t},a_{t})+\alpha H(\pi (\cdot |s_{t})))]$$

where J(π) denotes the expected return under the policy π, $\rho_{\pi }$ is the marginal distribution of state-action pairs under π, γ is the discount factor, $r(s_{t},a_{t})$ represents the instantaneous reward, $H(\pi (.|s_{t}))$ is the entropy regularization term, and α is the entropy weight coefficient.

In the SAC, three core functions need to be learned: the policy function $\pi (a_{t}|s_{t})$, the soft Q-value function $Q(s_{t},a_{t})$, and the soft state value function $V(s_{t})$. To achieve this, parameterized functions $Q_{\theta }(s_{t},a_{t})$ and $\pi_{\phi }(a_{t}|s_{t})$ are used, and their parameters θ and ϕ are alternately optimized via stochastic gradient descent. Below, the update rules for these parameters are explained in detail.

The training objective of the soft Q-value function is to minimize the soft Bellman residual, and its loss function is defined by Equation (13).

(13) $$L_{Q}(\theta )=E_{(s_{t},a_{t},r_{t},s_{t+1})∼D}\left[\frac{1}{2}{\left(Q_{\theta }(s_{t},a_{t})-\left(r(s_{t},a_{t})+\gamma E_{a_{t+1}∼\pi_{\phi }}\left[Q_{\bar{\theta }}(s_{t+1},a_{t+1})-\alpha \log\left(\pi_{\phi }(a_{t+1}|s_{t+1})\right)\right]\right)\right)}^{2}\right]$$

where $Q_{\bar{\theta }}$ is the target Q-value network. The parameters of the target Q-value network $\bar{\theta }$ are updated via an exponential moving average of θ, as shown in Equation (14), which has been proven to stabilize training [22].

(14) $$\bar{\theta }\leftarrow \tau \theta +(1-\tau )\bar{\theta }$$

where τ∈[0,1] is the update step size, controlling the smoothing degree.

The soft state value function $V(s_{t})$ is indirectly parameterized via the soft Q-value function and is optimized using stochastic gradient descent based on the objective, as defined by Equation (15).

(15) $$V(s_{t})=E_{a∼\pi_{\phi }}\left[Q_{\theta }(s_{t},a_{t})-\alpha \log\pi_{\phi }(a_{t}|s_{t})\right]$$

The policy function $\pi_{\phi }(a_{t}|s_{t})$, parameterized by the neural network with parameters ϕ, is trained by minimizing the expected Kullback–Leibler (KL) divergence. The objective is defined by Equation (16).

(16) $$L_{\pi }(\phi )=E_{s_{t}∼D}\left[E_{a_{t}∼\pi_{\phi }}\left[\alpha \log\left(\pi_{\phi }(a_{t}|s_{t})\right)-Q_{\theta }(s_{t},a_{t})\right]\right]$$

Moreover, the nature of the action space directly affects the form of the policy function. In this study, we define the load-shedding control in the power system as a continuous action space. Therefore, the policy function is modeled as a Gaussian distribution, as shown in Equation (17).

(17) $$\pi_{\phi }(a_{t}|s_{t})=N(\mu_{\phi }(s_{t}),\sigma_{\phi }(s_{t}))$$

where $\mu =\mu_{\phi }(s_{t})$ and $\sigma =\sigma_{\phi }(s_{t})$ represent the mean and standard deviation, respectively. μ and σ represent the outputs of the policy network. Based on the current state $s_{t}$, the stochastic policy $\pi_{\phi }$ generates an action distribution according to Equation (17) and samples the action $a_{t}$. To enable gradient backpropagation, the reparameterization trick is adopted. This reformulates the conditional Gaussian distribution into a function $a_{t}=f_{\phi }(s_{t},\xi )$, where ξ∼N(0,1). Specifically, the action $a_{t}$ is deterministically constructed by sampling ξ from a standard normal distribution and combining it with the policy network’s outputs:

(18) $$a_{t}=\mu +\xi .\sigma$$

Algorithm 1 provides the detailed implementation of the SAC algorithm.
**Algorithm 1.** Soft Actor-Critic (SAC)
1: Initialize the actor network $\pi_{\phi }$ randomly 2: Initialize the soft critic networks $Q_{\theta_{1}}$, $Q_{\theta_{2}}$ randomly 3: Initialize the target critic network $Q_{{\bar{\theta }}_{1}}$, $Q_{{\bar{\theta }}_{2}}$ with $Q_{\theta_{1}}$ and $Q_{\theta_{2}}$ 4: Initialize experience replay memory D, mini-batch size B 5: **for** each iteration **do**
6:     **for** each environment step **do**
7:         $a_{t}\sim \pi_{\phi }(a_{t}|s_{t})$ 8:         $s_{t+1}\sim p(s_{t+1}|s_{t},a_{t})$ 9:         $D\leftarrow D\cup \{(s_{t},a_{t},r(s_{t},a_{t}),s_{t+1})\}$ 10:       **end for** 11:       **for** each gradient step **do** 12:           $\theta_{i}\leftarrow \theta_{i}-\lambda_{Q}{\hat{\nabla }}_{\theta_{i}}L_{Q}(\theta_{i}),\;i\in \{1,2\}$ 13:           $\phi \leftarrow \phi -\lambda_{\pi }{\hat{\nabla }}_{\phi }L_{\pi }(\phi )$ 14:           ${\bar{\theta }}_{i}\leftarrow \tau \theta_{i}+(1-\tau ){\bar{\theta }}_{i},\;i\in \{1,2\}$ 15:       **end for** 16: **end for**


### 3.3. DRL Method Based on Graph Convolution

**Figure 1:**
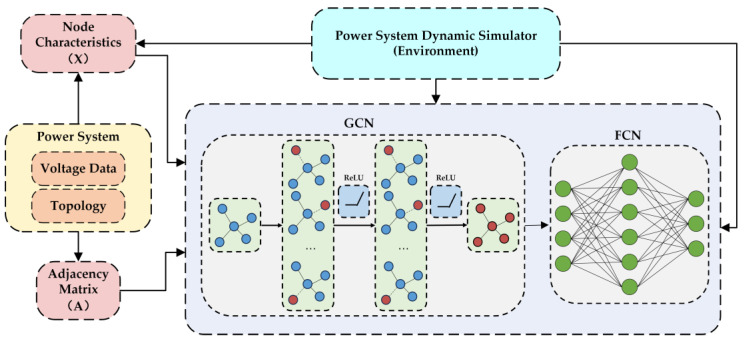
Reinforcement learning framework for power systems with GCN enhanced feature extraction.

In this section, we propose a novel graph convolutional network-based DRL algorithm, referred to as the GCN–SAC. This algorithm is specifically designed to adapt to the dynamic topological changes of power systems and effectively implement load-shedding strategies to address short-term voltage stability issues caused by FIDVR. Figure 1 provides an intuitive illustration of the overall architecture of this method within the reinforcement learning framework.

**Figure 2:**
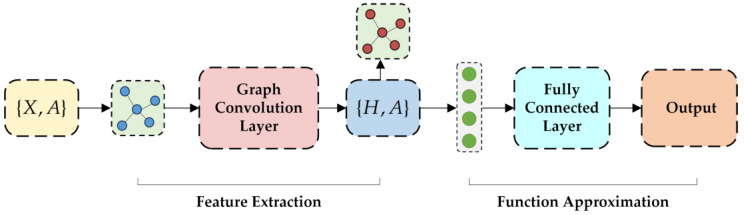
GCN enhanced feature extraction and functional approximation framework.

To facilitate implementation, the proposed algorithm is divided into two core modules: the feature extraction module and the function approximation module, as shown in Figure 2. The feature extraction module, implemented using a GCN, is primarily responsible for extracting topological information from the power system. The function approximation module, implemented with a fully connected network (FCN), focuses on generating the final decision outputs.

In the feature extraction module, we designed two layers of graph convolution, each followed by a ReLU activation function to ensure nonlinear representation capability. The output of the graph convolution layers is flattened into a one-dimensional feature vector and passed to the function approximation module. The function approximation module consists of two fully connected layers, each also followed by a ReLU activation function. Finally, the network’s output is used to generate the optimized decision.

The specific implementation process of the proposed GCN–SAC algorithm is detailed in Algorithm 2. The most challenging part of this algorithm lies in training the GCN layers as an independent feature extraction model. To address this, we tightly integrate the GCN model into the optimization loop by extending the classical Q-value network into a GCN-based Q-value network $Q^{GCN}(s_{t},a_{t})$. The detailed implementation process of the GCN–SAC is outlined in Algorithm 2.
**Algorithm 2.** GCN–SAC1: Initialize the actor network ${\pi_{\phi }}^{GCN}$ randomly2: Initialize the soft critic networks ${Q_{\theta_{1}}}^{GCN}$, ${Q_{\theta_{2}}}^{GCN}$ randomly3: Initialize the target critic network ${Q_{{\bar{\theta }}_{1}}}^{GCN}$, ${Q_{{\bar{\theta }}_{2}}}^{GCN}$ with ${Q_{\theta_{1}}}^{GCN}$ and ${Q_{\theta_{2}}}^{GCN}$
4: Initialize experience replay memory D, mini-batch size B
5: Initialize topological configurations $\left[T_{1},\ldots ,T_{p}\right]$
6: **for** episode n=1,M
**do**
7:    Randomly select a topology $T_{l}$ and update graph adjacency $A_{l}$
8:    $s\leftarrow s_{0}=\{X_{0},A_{l}\}$, initialize the environment with selected topology $T_{l}$
9:    **for**
t=1,T
**do**10:      Get charging decision $a_{t}$ according current $s_{t}$ using $\pi_{\phi }$
11:      Execute at and obtain reward $r_{t}$ and next state $s_{t+1}$
12:      Store the transition $\left\{s_{t},a_{t},r_{t},s_{t+1}\right\}$ into D
13:      Select random mini-batch of size B from D
14:     Update critic network weights using gradient in (13) 15:     Update actor network weights using gradient in (16) 16:     Update target critic network weights using Equation (14)17:   **end for**
18: **end for**


### 3.4. Modeling the MDP for Power Grid Emergency Control

In this section, we detail the definition of the state space, action space, and reward function based on the physical characteristics of the power grid emergency control problem. These definitions provide the theoretical basis and optimization objectives for the agent’s learning process.

1. State

The environmental state is considered the input to the control strategy, which is used to generate the corresponding actions. In this study, the state is defined as the combination of the node voltages and the power system topology at time t, where the node voltages include historical information from the past T time steps. This state representation simultaneously captures the system’s dynamic changes and topological characteristics, providing comprehensive input information for strategy optimization. The specific definition is shown in Equation (19).

(19) $$s_{t}=\{X_{t},A_{t}\}$$

where $X_{t}$ is defined in Equation (11) and $A_{t}$ represents the topological connections of the power system.

2. Action

The action $a_{t}$ represents the load-shedding amount corresponding to a given state $s_{t}$ in the power system. Specifically, $a_{t}$ is defined as a continuous value indicating the proportion of the load to be shed. The action space is defined, as shown in Equation (20).

(20) $$a_{t}\in [0,1]$$

where $a_{t}=0$ indicates no load shedding in the current state, and $a_{t}=1$ indicates shedding the entire load (i.e., a load-shedding proportion of 100%).

3. Reward

**Figure 3:**
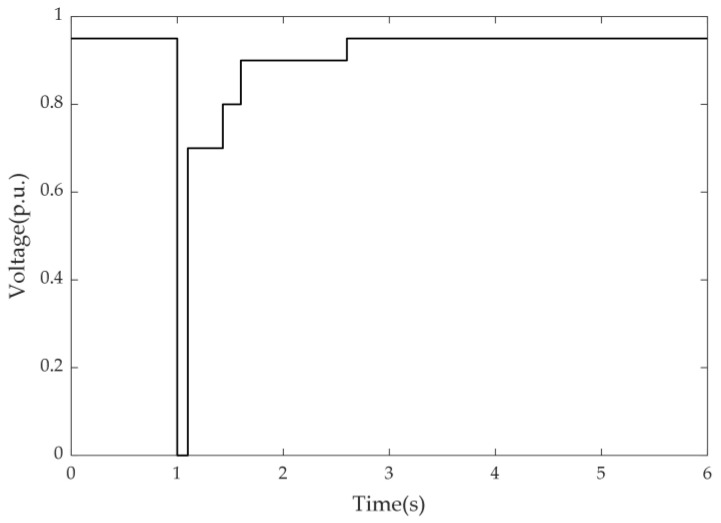
Transient voltage recovery standard for transmission systems.

The reward $r_{t}$ represents the immediate feedback signal received when the system transitions from state $s_{t}$ to state $s_{t+1}$ after performing action $a_{t}$. It is used to guide the agent in optimizing its control strategy. In this study, we refer to the standards proposed in [23] (as shown in Figure 3) to define the reward function. The specific form of the reward function is given by Equation (21).

(21) $$r_{t}=\left\{\begin{array}{l} -20000,\;v_{i}(t)<0.95,t>T_{pf}+4\\ c_{1}R_{volt}-c_{2}R_{shed}-c_{3}R_{invalid},\;otherwise \end{array}\right.$$

where $T_{pf}$ is the time when the fault is cleared, and $c_{1}$, $c_{2}$ and $c_{3}$ are weight factors. The reward function, as described in Equation (21), comprises three penalty components, which are explained as follows:

- Voltage deviation penalty ($R_{volt}$): This penalty measures the sum of deviations between bus voltages and standard voltage thresholds, encouraging the agent to minimize voltage deviations and maintain system stability. It is calculated as follows:

(22) $$R_{volt}=\sum_{i=1}^{N}\Delta v_{i}(t)$$

(23) $$\Delta v_{i}(t)=\left\{\begin{array}{l} \min\{v_{i}(t)-0.7,0\},T_{pf}<t\leq T_{pf}+0.33\\ \min\{v_{i}(t)-0.8,0\},T_{pf}+0.33<t\leq T_{pf}+0.5\\ \min\{v_{i}(t)-0.9,0\},T_{pf}+0.5<t\leq T_{pf}+1.5\\ \min\{v_{i}(t)-0.95,0\},T_{pf}+1.5<t \end{array}\right.$$

where $v_{i}$ represents the voltage magnitude of bus i, and N denotes the total number of buses in the system.

- Load shedding penalty ($R_{shed}$): This penalty represents the total load shed across the system, encouraging the agent to minimize load shedding while ensuring grid stability. It is computed as follows:

(24) $$R_{shed}=\sum_{j=1}^{M}\Delta L_{j}$$

where $\Delta L_{j}$ represents the load shedding amount at bus j, and M denotes the number of buses participating in load shedding.

- Invalid action penalty ($R_{invalid}$): This penalty is applied when the agent issues a load-shedding command for a bus where the load has already been fully shed, discouraging invalid actions. It is calculated as follows:

(25) $$R_{invalid}=\sum_{k=1}^{M}I(L_{k}^{\left(t-1\right)}=0\wedge a_{k}>0)$$

where $I\left(.\right)$ is an indicator function that equals 1 when the condition is true, and 0 otherwise, $L_{k}^{\left(t-1\right)}$ represents the remaining load at bus k at time t−1, and $a_{k}$ denotes the load shedding action taken by the agent at bus k.

Additionally, to further reinforce system stability constraints, a global constraint mechanism is incorporated into the reward function: if the voltage at any bus falls below 0.95 p.u. within 4 s after fault clearance, the reward function is forcibly set to a significant negative value (−20,000) to severely penalize actions that fail to maintain system voltage stability.

## 4. Simulation

**Figure 4:**
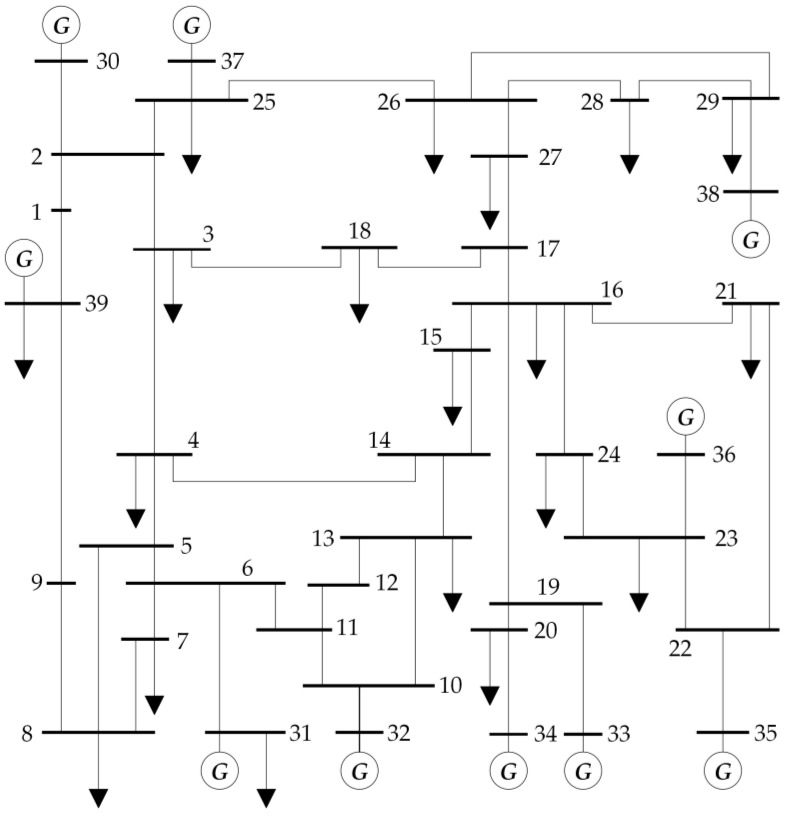
The topology of the IEEE 39-bus system.

To verify the correctness and feasibility of the proposed method, this study uses the IEEE 39-bus test system (with its network topology shown in Figure 4) as an example to conduct a comprehensive simulation analysis of the GCN–SAC algorithm. In terms of performance comparison, we incorporated mainstream reinforcement learning algorithms (SAC, DDPG, and PPO) as well as the traditional UVLS relay control scheme. Through a multidimensional comparative analysis, we systematically evaluated the proposed method’s performance in voltage recovery effectiveness and load shedding strategies. This chapter first introduces the simulation setup, providing a detailed description of the experimental parameters and related assumptions. Subsequently, the training process of the GCN–SAC is compared with that of the SAC algorithm to analyze the role of GCNs in enhancing state representation and improving training efficiency. A systematic assessment of the training and testing performance of the GCN–SAC algorithm is also conducted. Finally, focusing on the FIDVR fault scenario, a detailed comparison of the five methods in terms of voltage recovery performance and load shedding strategies is presented. The evaluation comprehensively examines the proposed method’s advantages from multiple perspectives, including voltage stability and load shedding cost, thereby verifying its superiority and practicality in addressing complex disturbance scenarios.

### 4.1. Simulation Setup

The OpenAI Baselines framework was used to learn a closed-loop control strategy with the goal of preventing FIDVR by implementing load shedding at buses 3, 16, and 28, while simultaneously meeting the voltage recovery requirements shown in Figure 3. All experiments were conducted on a computer equipped with an Intel Core i7-14700F processor (manufactured by Intel Corporation, Santa Clara, CA, USA) and an NVIDIA RTX 4060 GPU with 8GB GDDR6 VRAM (manufactured by NVIDIA Corporation, Santa Clara, CA, USA).

The observation metrics include the voltage magnitudes at buses 3, 16, and 28, as well as the corresponding changes in their loads. In the experiments, the parameter T in Equation (10) was set to 20. Based on a comprehensive trade-off analysis of system performance, the weight coefficients in the reward function of Equation (21) were set as follows: $c_{1}=275$, $c_{2}=180$, and $c_{3}=6$. 

**Figure 5:**
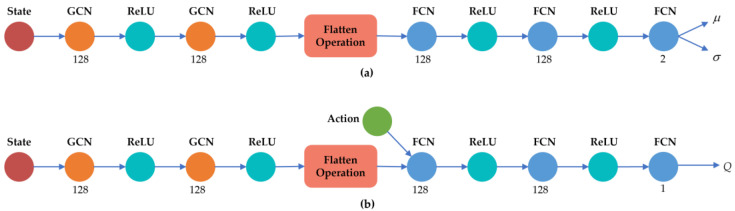
The policy and value network architecture of the GCN–SAC algorithm: (**a**) network architecture of the policy network, (**b**) network architecture of the value network.

For the proposed GCN–SAC method, the architecture of the policy network and value network is shown in Figure 5. Both networks consist of two graph convolutional layers, each containing 128 neurons, followed by two fully connected layers, also with 128 neurons per layer. The output of the graph convolutional layers is flattened and passed to the fully connected layers, culminating in the output layer to generate the final results.

The key hyperparameters are set as follows: the total number of episodes during training is 500,000, and the learning rate for the GCN component is 0.00005. Within the SAC framework, the learning rates for both the actor and critic networks are set to 0.0001, the discount factor γ is 0.99, the soft update coefficient τ is 0.005, the batch size is 64, and the capacity of the experience replay buffer is 50,000.

### 4.2. Training and Testing Performance

During the training process, each episode starts from a dynamically stable simulation state. At 1.0 s of simulation time, a short-circuit fault is randomly applied to bus 3, 15, or 27. The fault duration is randomly set to 0.0 s (no fault), 0.05 s, or 0.1 s, and the fault is designed to clear automatically. To modify the system topology, the following four configurations were implemented:

- No lines disconnected.
- Disconnection of the line between bus 1 and bus 2.
- Disconnection of the line between bus 15 and bus 16.
- Disconnection of the line between bus 26 and bus 27.

**Figure 6:**
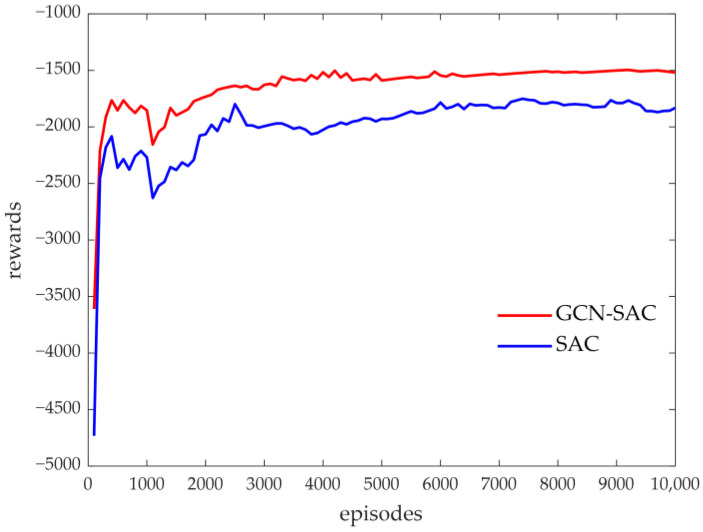
The moving average rewards during the DRL training.

For the same training scenario, the performance of the GCN–SAC and SAC was compared. In the design of the SAC algorithm, the $X_{t}$ and $A_{t}$ matrices were vectorized, and the state space was set to $s_{t}=\{vec(X_{t}),vec(A_{t})\}$. Figure 6 illustrates the moving average of rewards during the first 10,000 episodes of training for both the GCN–SAC and the SAC. As shown in the figure, the GCN–SAC demonstrates a faster and more stable convergence performance.

To assess the trained agent’s transfer learning capabilities in unexpected settings, the testing process included short-circuit defects at all 39 buses, with three different fault durations randomly set (i.e., 0.03 s, 0.05 s, and 0.1 s). For each fault location and duration, 20 different topology configurations were designed, resulting in a total of 39 × 20 = 780 test cases. The performance of the algorithm during the testing phase was measured by comparing the reward differences among these test cases. Out of the 780 test scenarios, 476 were likely to trigger FIDVR issues without any control measures, necessitating load-shedding operations.

**Figure 7:**
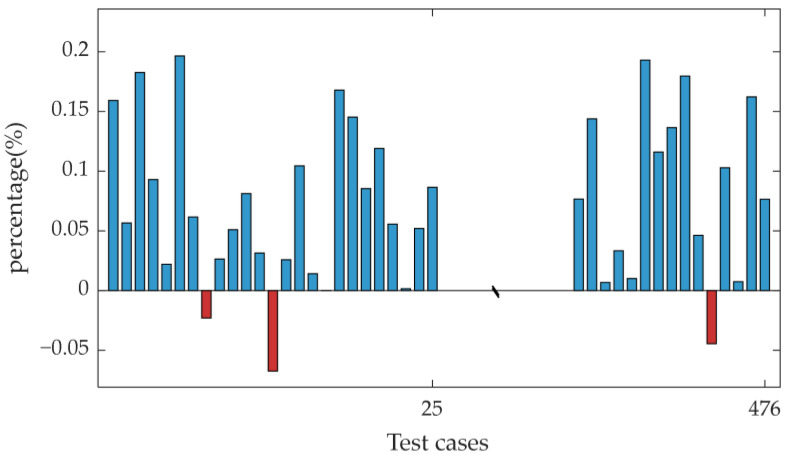
Histogram of reward differences between the GCN–SAC and SAC for 476 test cases.

To visually present the performance comparison results, the reward differences between the GCN–SAC and the SAC were calculated for all test scenarios requiring load shedding (i.e., the GCN–SAC reward minus the SAC reward). A positive value indicates that the GCN–SAC method outperformed the SAC method in the corresponding scenario. Figure 7 displays the histogram illustrating the differences in rewards between the GCN–SAC and SAC methods, revealing that the GCN–SAC outperformed the SAC in 91.78% of the test scenarios.

The average computation time for a single action decision step using the GCN–SAC and SAC methods was 0.78 ms and 0.74 ms, respectively. During a 10-s simulation event, the total computation time for the GCN–SAC method averaged 0.078 s, demonstrating its capability to meet real-time operational requirements.

### 4.3. Load Shedding Strategy

**Figure 8:**
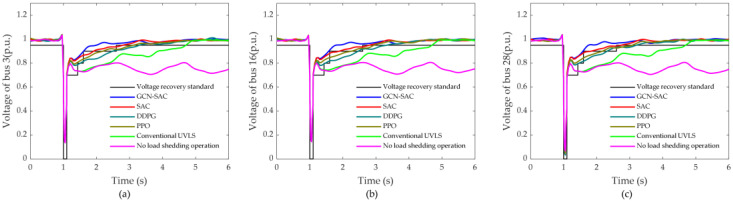
Voltage recovery curves and voltage recovery criteria for different load shedding control schemes: (**a**) bus 3; (**b**) bus 16; (**c**) bus 28.

Table 1 and Figure 8 present a performance comparison of the GCN–SAC, SAC, DDPG, PPO, and the traditional UVLS relay control scheme under a specific test scenario. In this scenario, a fault is assumed to occur at bus 26 with a duration of 0.1 s. To enhance the realism and robustness of the test, an additional 1% Gaussian noise is introduced into the observation data.

Table 1 demonstrates that, compared to the traditional UVLS relay control method, deep reinforcement learning (DRL)-based control methods exhibit significant advantages in load-shedding performance. Specifically, the GCN–SAC approach outperforms the SAC method in load-shedding strategies at bus 3 and bus 16. Furthermore, both the GCN–SAC and SAC methods achieve markedly better load-shedding performances at buses 3, 16, and 28, compared to the DDPG and PPO methods. Additionally, as shown in Figure 8, DRL-based methods exhibit superior voltage recovery performance compared to the traditional UVLS relay control method. Specifically, the UVLS relay method fails to restore the voltage to standard levels within 4 s after fault clearance, resulting in greater load shedding at buses 3, 16, and 28. Under the same test scenario, the total reward of the GCN–SAC is −1518.6, significantly outperforming other algorithms, indicating its ability to quickly restore system stability with minimal load-shedding cost. By contrast, the SAC method achieves a total reward of −1831.3, which, although inferior to the GCN–SAC, remains superior to the DDPG and PPO. The total rewards for the DDPG and PPO are −2054.2 and −1892.6, respectively, reflecting their relatively weaker optimization capabilities in addressing complex voltage instability problems. Notably, the highest reward value of the GCN–SAC demonstrates its effectiveness in achieving voltage recovery targets while significantly reducing the amount of load shedding. Conversely, the lower reward values of the SAC, DDPG, and PPO indicate their reliance on greater load-shedding efforts to restore voltage. In summary, the GCN–SAC algorithm surpasses other methods in terms of reward value, load-shedding proportion, and voltage recovery performance, showcasing its exceptional advantages.

Through comparative analysis of different algorithms, it can be concluded that the GCN–SAC algorithm performs the best. This superiority is attributed to its effective integration of graph neural networks for precise modeling of the power system topology and the SAC algorithm’s efficient learning capabilities in continuous action spaces, thereby achieving more optimized control strategies.

## 5. Conclusions

This paper proposes a novel DRL approach that combines a Graph Convolutional Network (GCN) with the soft actor-critic (SAC) algorithm, addressing the challenge of voltage control in smart grids through load shedding. Experimental results demonstrate that the proposed method excels in extracting grid topology features and optimizing continuous action strategies. Simulation evaluations based on the IEEE 39-bus system indicate that the approach significantly enhances voltage recovery with minimal load shedding under complex load disturbances and dynamic topology changes, thereby ensuring stable grid operation. Compared to existing methods, the proposed approach exhibits stronger adaptability and control precision when handling various operational scenarios and uncertain disturbances, highlighting its robustness. The innovation of this study lies in the first-ever integration of a GCN’s topological embedding capability with the SAC’s reinforcement learning features, providing a novel technical solution to the load-shedding optimization problem.

Despite the promising results achieved in this study, there remains room for improvement in enhancing the practical application value and broader applicability of the proposed method. Firstly, regarding scalability and computational efficiency in large-scale power grids, while the simulation results on the IEEE 39-bus system validate the effectiveness of the method, its applicability to larger-scale grids requires further evaluation. Future research will involve more complex test systems, such as the IEEE 118-bus system or actual large-scale grid models, to systematically assess the method’s performance in handling higher-dimensional topological features and complex disturbances. Additionally, optimization of model training and inference efficiency—through approaches such as the integration of distributed computing frameworks and lightweight neural network structures—will ensure the method’s real-time capability in large-scale systems, thereby further improving its practicality. Secondly, in terms of multi-objective optimization and adaptability to real-world applications, this study primarily focuses on voltage control and load shedding, without fully considering practical operational objectives, such as economic efficiency and renewable energy integration. Future work will aim to incorporate economic indicators (e.g., operational costs and load-shedding expenses) and renewable energy utilization goals into a multi-objective optimization framework. This approach will enable dynamic trade-offs between load-shedding costs, voltage stability, and clean energy utilization, aligning the proposed method more closely with the operational needs of modern power grids, particularly in scenarios with high penetration of renewable energy. Lastly, to further enhance the method’s applicability and robustness, future research will extend to more complex real-world scenarios, including distributed energy resource integration, dynamic load behavior, and complex grid topologies. Validation under diverse operating conditions will enable a comprehensive exploration of the method’s performance in addressing nonlinear dynamic disturbances, system faults, and multi-time-scale responses. These efforts aim to provide a solid theoretical foundation and technical support for the practical deployment of the proposed method in smart grids.

In summary, the GCN–SAC joint optimization method proposed in this paper offers an innovative and efficient solution to voltage control challenges in modern smart grids. The research results demonstrate that the proposed method offers significant advantages in ensuring system stability and optimizing load-shedding control, while also providing valuable support and insights for technological advancements and practical applications in the smart grid domain. In the future, through further expansion and refinement in large-scale power grids, multi-objective optimization, and complex real-world scenarios, this method is expected to contribute substantially to the development of a low-carbon, high-efficiency modern power grid.

## Figures and Tables

**Table 1:** Comparison of different solutions for bus load shedding (Bold values in the table indicate the method with the lowest load shedding rate among the solutions).

Bus Number	Control Scheme	Initial Load (MW)	Load Reduction (MW)	Load Reduction Ratio
bus 3	GCN–SAC	200	58.52	**0.2926**
	SAC	200	66.37	0.3319
	DDPG	200	77.64	0.3882
	PPO	200	73.09	0.3655
	UVLS	200	102.71	0.5136
bus 16	GCN–SAC	150	47.27	**0.315** **1**
	SAC	150	71.84	0.4789
	DDPG	150	78.30	0.5220
	PPO	150	76.45	0.5097
	UVLS	150	83.27	0.5551
bus 28	GCN–SAC	120	43.78	0.3648
	SAC	120	43.74	**0.3645**
	DDPG	120	47.63	0.3929
	PPO	120	45.95	0.3829
	UVLS	120	65.66	0.5472

## Data Availability

The data used in this study are sourced from the MATPOWER toolbox, a publicly available and widely used open-source resource for power system analysis. The specific test case utilized is the IEEE 39-bus system (case39), which is included in the MATPOWER distribution. MATPOWER can be accessed freely at https://matpower.org, accessed on 19 November 2024.

## **Abbreviations**

The following abbreviations are used in this manuscript:

DRL	Deep Reinforcement Learning
GCN	Graph Convolutional Network
SAC	Soft Actor-Critic
MDP	Markov Decision Process
RL	Reinforcement Learning
DDRL	Distributed Deep Reinforcement Learning
FIDVR	Fault-Induced Delayed Voltage Recovery
DDPG	Deep Deterministic Policy Gradient
TD3	Twin Delayed Deep Deterministic Policy Gradient
PPO	Proximal Policy Optimization
FCN	Fully Convolutional Network
ReLU	Rectified Linear Unit
UVLS	Under Voltage Load Shedding
AI	Artificial Intelligence
EPSO	Evolutionary Particle Swarm Optimization
MADRL	Multi-Agent Deep Reinforcement Learning
PARS	Power-Adaptive Reinforcement Search
